# Impact of Sleep Fragmentation on Cognition and Fatigue

**DOI:** 10.3390/ijerph192315485

**Published:** 2022-11-22

**Authors:** Oumaïma Benkirane, Bérénice Delwiche, Olivier Mairesse, Philippe Peigneux

**Affiliations:** 1 UR2NF—Neuropsychology and Functional Neuroimaging Research Unit, at CRCN—Centre for Research in Cognition and Neurosciences and UNI—ULB Neuroscience Institute, Université Libre de Bruxelles (ULB), 1050 Brussels, Belgium; 2 Brugmann University Hospital, Sleep Laboratory & Unit for Chronobiology U78, Université Libre de Bruxelles (ULB), 1050 Brussels, Belgium

**Keywords:** sleep fragmentation, cognitive fatigue, cognitive functions

## Abstract

Sleep continuity and efficacy are essential for optimal cognitive functions. How sleep fragmentation (SF) impairs cognitive functioning, and especially cognitive fatigue (CF), remains elusive. We investigated the impact of induced SF on CF through the TloadDback task, measuring interindividual variability in working memory capacity. Sixteen participants underwent an adaptation polysomnography night and three consecutive nights, once in a SF condition induced by non-awakening auditory stimulations, once under restorative sleep (RS) condition, counterbalanced within-subject. In both conditions, participants were administered memory, vigilance, inhibition and verbal fluency testing, and for CF the TloadDback, as well as sleep questionnaires and fatigue and sleepiness visual analog scales were administered. Subjective fatigue increased and sleep architecture was altered after SF (reduced sleep efficiency, percentage of N3 and REM, number of NREM and REM phases) despite similar total sleep time. At the behavioral level, only inhibition deteriorated after SF, and CF similarly evolved in RS and SF conditions. In line with prior research, we show that SF disrupts sleep architecture and exerts a deleterious impact on subjective fatigue and inhibition. However, young healthy participants appear able to compensate for CF induced by three consecutive SF nights. Further studies should investigate SF effects in extended and/or pathological disruption settings.

## 1. Introduction

Uninterrupted and qualitative night-time sleep plays a key role in physical regeneration and the maintenance of optimal brain and bodily functions [1,2,3]. Conversely, sleep deprivation (SD) or impaired sleep quality can exert a deleterious impact on both physical health and cognitive performance [4,5]. Notwithstanding a wide interindividual variability in sleep requirements [6], it is estimated that most adults need an average 7 h of sleep per night to avoid negative health effects [7]. Still, sleep may not be efficient even in individuals who experience nights of normal duration [8], potentially due to various factors that can affect sleep quality and its continuity [9]. Regarding continuity, sleep fragmentation, characterized by repeated episodes interrupting sleep that do not systematically lead to awakenings but prevent the brain from entering consolidated sleep stages, was shown to impair sleep restorative effects [10] and exert deleterious consequences on daytime cognitive functions [11,12].

Sleep fragmentation is a major component of sleep-related breathing disorders (SRBD) and especially Obstructive Sleep Apnoea (OSA), defined by recurrent episodes of airflow obstructions, eventually resulting in brief arousals, intermittent hypoxemia, snoring and sleep fragmentation [13]. While some studies have shown a specific effect of sleep fragmentation on cognitive performance in OSA patients [14], there is still an ongoing debate about the respective impacts of hypoxemia and sleep fragmentation on diurnal neurocognitive deficits [14,15,16]. Nonetheless, sleep fragmentation is at least an aggravating component in OSA-related disturbances. Indeed, neurocognitive deficits are frequently reported in OSA conditions in which sleep restorative effects are impaired [17,18]. Deficits are mostly prevalent in tasks that require a continuous use of cognitive resources (e.g., sustained attention and executive functions), but SRBD can also exert a deleterious impact on various aspects of memory, productivity, and social interactions [19,20,21,22,23]. Additionally, in healthy sleepers, noise-prompted sleep fragmentation enhances upper airway collapsibility [24] and heart rate [25] that are typical of OSA [26]. Since the independent contributions of sleep fragmentation and hypoxemia to these cognitive deficits are still under scrutiny [14], investigating the consequences of sleep fragmentation on cognitive function in healthy sleepers is an important step in further understanding the impact of the disruption of normal restorative sleep mechanisms on cognition in a clinical model, such as patients with OSA. Hence, systematically investigating the impact of experimentally induced and reversible sleep fragmentation in healthy populations may be a sensible experimental model to investigate how sleep fragmentation contributes to OSA-related cognitive deficits.

In particular, cognitive fatigue (CF) is a symptom that can be linked to both sleep fragmentation and SRBD, in which it is subjectively experienced above clinical thresholds next to excessive daytime sleepiness [27]. Mental or cognitive fatigue (CF) can be defined as a decrease in cognitive efficiency developing with sustained cognitive demands in constrained processing time conditions, independently of sleepiness [28]. CF might result from extended duration and/or restricted processing time on mentally demanding tasks, eventually generating subjective fatigue, perceptions of tiredness and lack of energy and performance decline [29]. Past CF studies mostly used tasks in which a constant cognitive requirement (e.g., mental arithmetic calculations) was held for a long duration, up to hours [30,31,32,33,34,35,36], and/or under high cognitive task demands, eventually leading to increased CF. The underlying postulate is that sustained cognitive solicitations (e.g., in a demanding working memory updating task) will deplete cognitive resources and thus give rise to higher CF levels [37,38]. Cognitive fatigue can be also conceptualized within the framework of the time-based resource-sharing (TBRS [39]) model, as proposed by Borragan et al. [28]. In this framework, it is not task complexity in itself that leads to cognitive load and eventually fatigue, but the time allowed to process the material, attention being a finite resource that varies between individuals. Hence, a personal maximal cognitive load corresponds to the fastest pace at which an individual still accurately meets task demands. Working continuously at this maximal cognitive load will ultimately result in increased CF. Using the TloadDback task, a cognitive load task specifically designed to account for interindividual variability in working memory processing capabilities, CF was found to be modulated by cognitive load levels [28], as well as sleep deprivation [40] and duration [41]. How sleep fragmentation contributes to CF and impairs cognitive functioning, however, remains barely investigated.

In the present study, we investigated the impact on young healthy participants of experimentally induced sleep fragmentation (SF) mimicking OSA-related interruptions during three consecutive nights on CF and neurocognitive functions, as compared to three nights spent in the laboratory under normal restorative sleep (RS) conditions. Participants underwent polysomnography (PSG), were administered a neuropsychological battery covering the main cognitive functions, and were exposed to a CF-inducing dual-working-memory updating task (TloadDback [28]) under low and high cognitive demand conditions. With this within-subject design, we aimed to understand the relationships between subjective and objective markers of fatigue and their relationship with cognitive performance, and the impact of the SF-induced disruption of normal restorative sleep mechanisms on the induction of CF and cognitive abilities.

## 2. Materials and Methods

Participants

Sixteen healthy good sleepers (8 females, 8 males, mean age 28.5 ± 4.48 years, minimal age = 24 years, maximal age = 38 years) were recruited using social media advertisements and flyers. Participants were naïve to the intent of the experiment and gave written informed consent to participate in this study, approved by the Faculty and ULB-Erasme hospital ethics committees (CE 001/2019). They received EUR 250 for their participation. Exclusion criteria were sleep or breathing disorders, irregular sleep–wake schedule, extreme morning or evening chronotype, usual sleep duration shorter than 6.5 h, neurological or psychiatric conditions, history of opioid treatment or current benzodiazepine intake. Participants were also asked to avoid stimulating and/or alcoholic drinks the day before as well as during the experimental days. Additionally, absence of sleep or breathing disorder was controlled during a habituation PSG night spent in the sleep laboratory one week prior to start the experiment. Regularity of the sleep–wake cycle and sufficient sleep duration was controlled with 7 days of actigraphic monitoring and completion of a daily sleep agenda prior to the first testing day (see details below).

Experimental procedure

**Figure 1:**
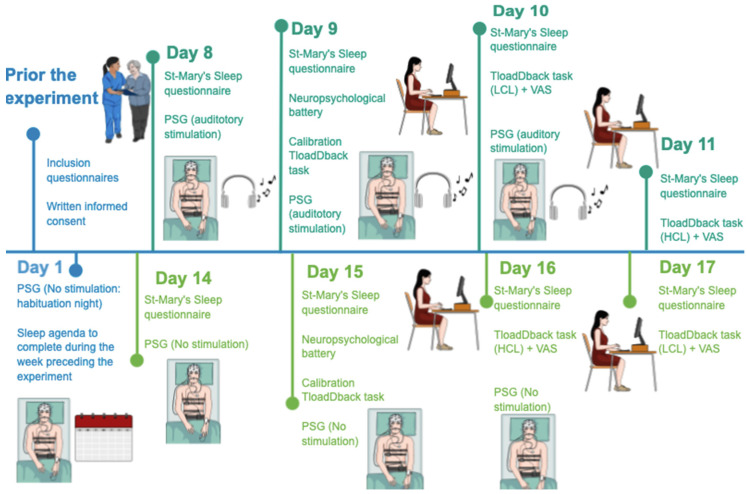
Experimental protocol. Note: PSG = Polysomnography. VAS = Visual Analogue Scales (fatigue, sleepiness, stress, and motivation). LCL = Low Cognitive Load Condition. HCL = High Cognitive Load Condition.

The experiment spanned over 17 days per participant (Figure 1). The first habituation night was spent in the sleep laboratory to ensure the absence of sleep and breathing disorders and habituate to sleep under PSG. Participants returned home in the morning for the week. To ensure sleep–wake regularity during that period at home, the participants were asked to fill in a sleep agenda every morning and wear an actigraphy device (wGT3X-BT Monitor, ActiGraph, Pensacola, FL, USA). On day 8, they came back to the sleep laboratory for the first experimental night. The experimental manipulation consisted of 3 consecutive PSG nights spent in the sleep laboratory under RS (vs. SF) condition, then 3 nights of sleep at home, and then again 3 consecutive PSG nights in the sleep laboratory under SF (vs. RS) conditions. The order of RS and SF conditions was counterbalanced between participants, 8 beginning in the SF condition and 8 beginning in the RS condition. On all SF and RS experimental nights, participants arrived in the lab between 21:30 and 22:00, were prepared for PSG recording, and light-off time was set between 23:00 and 24:00 depending on individual habits; light-on time was set accordingly to each participant’s usual number of hours of sleep (mean 531.43 ± 58.82 min). In the SF condition, auditory stimuli were presented during the 3 consecutive nights to induce sleep fragmentation (see below). In the RS condition, they slept undisturbed. In the morning following each night in RS and SF conditions (days 9 to 11 and days 15 to 17), participants completed the St Mary’s Sleep Questionnaire [42], subjectively assessing the quality of the preceding night.

After the first RS and SF nights (days 9 and 15), participants were administered a neuropsychological assessment battery and the calibration part of the TloadDBack, aimed at determining their maximal working memory processing capacity (see below) [28]. After the second and third RS and SF nights (days 10–11 and 16–17), participants were administered the TloadDBack CF-induction protocol under either high (HCL) or low (LCL) cognitive conditions each day, in a within-subject, counterbalanced design. Participants were allowed a quiet resting period before, during and after the TloadDBack practice. CF was subjectively assessed using visual analog fatigue (VASf [43]) and sleepiness (VASs [44]) severity scales at each experimental step (T.I, T.II and T.III), as well as visual analog scales for stress (VASst) and motivation to control for potential confounds. Interindividual circadian variability was controlled for by testing each participant at the same time of the day in all RS and SF, LCL and HCL conditions.

Inclusion questionnaires

Participants completed questionnaires online prior to their inclusion in the experiment. Sleep quality (Pittsburgh Sleep Quality Index, PSQI [45]; cut-off score ≤ 6) and perceived impact of fatigue (Fatigue Severity Scale, FSS [46]; cut-off score < 4) were assessed for the previous month, as well as physical and mental fatigue (Brugmann Fatigue Scale, BFS [27]; cut-off score ≤ 6), circadian typology preference and strength (Chronotype Questionnaire, ChQ [47]; cut-off score distinctness (strongly marked/less flexible preference) ≥ 23), depression (Beck Depression Inventory, BDI [48]; cut-off score < 8), and anxiety (Beck Anxiety Inventory [49], cut-off score ≤ 35).

Experimental questionnaires

*Sleep Diaries*

Starting the morning following the habituation night and during the whole week preceding the beginning of the experiment, participants completed a sleep agenda (see Supplementary Materials for a template) enabling us to calculate subjective sleep onset latency, wake after sleep onset, sleep efficiency, total sleep time and sleep and wake quality. Every morning throughout the entire experimental period in each sleep condition (2 × 3 times), subjects completed the St Mary’s Sleep Questionnaire [42], comprising 14 questions subjectively assessing the quality of the preceding night.

*TloadDback-related Visual Analog Scales*

As stated above, 10 cm long visual analog scales (VAS) were administered on the second and third day of the RS and SF experimental sessions immediately before and after the TloadDback task, and 5 min later after the second NIRS/EEG resting state. At each step, 4 VAS assessed for fatigue (VASf [43]; from perfectly rested to completely exhausted), sleepiness (VASs [44]; from very alert/vigilant to very sleepy), stress (VASst; from not at all stressed to very stressed) and motivation (VASm; from not at all motivated to highly motivated). To control for interindividual differences in the subjective conceptions of fatigue and sleepiness, cognitive fatigue was described to the participants as “the necessity to cease persistent cognitive efforts without the urge to fall asleep”, while sleepiness was defined as “an intermediate state between waking and sleeping characterized by a tendency to doze off”.

Neuropsychological Evaluation

Neuropsychological tests covering verbal and visual short-term and working memory, episodic memory, attentional and executive functions were administered after the first experimental night in each RS and SF condition.

Episodic memory was assessed using the «RLS-15» [50], in which participants must remember a list of 15 words. After each presentation of the list, participants were asked to recall it until they were able to reproduce the entire list twice in succession (immediate recall with a maximum of 10 trials) followed by a delayed recall (RD) 30 min later. Outcome measures were the average of words correctly retrieved during the immediate recall trials (RM), the percentage of words recalled in at least two consecutive immediate recall trials from the total number of words recalled (% RLTC), and the number of correct words at delayed recall (RD).

During the interval between immediate recalls and the delayed RLS-15 recall, several tasks were performed. Verbal and visuo-spatial short-term memory performance was assessed using the digit span (subtest of the WAIS [51]) and block tapping [52] tests, respectively. Span performance was the longest series of numbers or positions repeated twice in the correct order. Working memory performance was assessed asking participants to reproduce digits in the reverse order (subtest of the WAIS [51]). Span performance was the longest series of numbers repeated twice in reverse order. Vigilance was assessed using the Psychomotor Vigilance Task (PVT [53]). Inhibition was assessed using the Stroop (French version [54]) task with 3 conditions: (1) coloured rectangles to name, (2) name of colours to read, and (3) colours to denominate based on the print colours without taking into account the written word. Outcome variables were completion times and number of corrected and uncorrected errors. Following the delayed recall in the RLS-15, verbal phonological and semantic fluency [54] were administered: participants had to produce as much words as possible in two minutes. In a counterbalanced design, for phonological fluency, words had to begin with the letters “p” or “o”, and for semantic fluency, words had to be included in the category of animals or fruits.

Cognitive Fatigue-Inducing TloadDback Task

The TloadDback task is a dual task combining a classical N-back working memory-updating task and odd/even number decision task [28]. Combining two tasks with different information processing requirements entails a sustained recruitment of working memory resources, modulated by the speed at which information needs to be processed. The task is described in detail elsewhere (Ref. [28]; Study 1); only essential information is provided here. In the TloadDback task, 30 digits and 30 letters per block are displayed on the screen in alternation (e.g., N–2–X-7–X–1–L…). Participants must alternatively (a) press the space key with their left hand every time the displayed letter is the same as the previous letter (1-back task; e.g., … X-7–X…), and (b) indicate whether the displayed digit is odd or even by pressing the “2” or “3” keys with their right hand on the numeric keypad. Recruitment of cognitive resources is individually tailored by adjusting, during a calibration session, the fastest item presentation speed (i.e., interstimulus interval, ISI) at which the participant is still able to successfully perform (i.e., average accuracy per block > 85%, weighted 65% for the 1-back component and 35% for the odd/even decision component).

In the calibration session after the first SF or RS night, participants were first familiarized with the 1-back and odd/even decision tasks independently then combined. They were then administered the TloadDback task for a maximum of 20 blocks, with a comfortable ISI of 1500 ms for the first block. Whenever performance for a block was ≥85%, the ISI for the next block was set at the prior ISI minus 100 ms, making the task more difficult as a shorter processing time was allowed. Blocks were administered using this staircase procedure until a score < 85% was achieved over three blocks, meaning that the participant’s cognitive load limit was reached.

**Figure 2:**
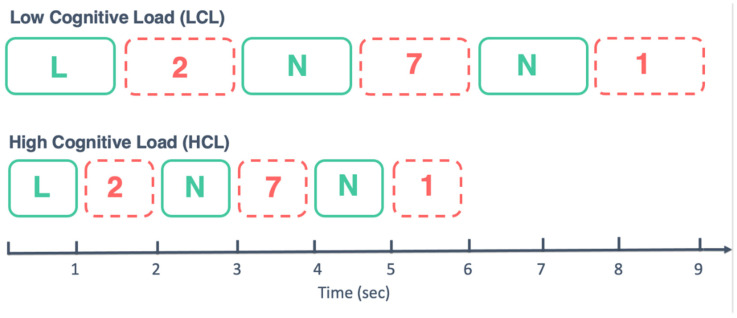
Course of the TloadDback task in both cognitive load conditions. ISI (interstimulus interval) in the LCL condition was determined as 1/3 longer than in the HCL condition (i.e., ISI (LCL) = ISI (HCL) + 1/2 ISI (HCL)).

Based on the outcome of the calibration, participants were administered the TloadDback for 16 min on the second and third days of the SF or RS experimental conditions under either a high (HCL) or a low (LCL) cognitive load condition. This procedure has been shown efficient to induce high vs. low levels of CF in healthy participants [55], and therefore counterbalance two conditions of a task permitting to better assess the potential links between sleep fragmentation (the expression of CF being partly contingent upon prior sleep conditions), and cognitive performance. In the HCL condition, the ISI was fixed for all blocks to the last successful ISI in the calibration session, increased by 100 ms. ISI in the LCL condition was determined as 1/3 longer than in the HCL condition (i.e., ISI (LCL) = ISI (HCL) + 1/2 ISI (HCL)). Therefore, LCL and HCL conditions share an equivalent level of complexity, whereas the ISI was proportionally distinct and customized to each participant’s maximal processing capability. Individual calibration for maximal processing capacity ensured that, in a HCL condition, the task will be challenging even for the candidates with high innate cognitive ability levels. Of note, since the ISI is different between individuals and HCL and LCL conditions, but the task is always stopped after 16 min practice, the number of processed blocks differed as a function of the ISI used (Figure 2). Cognitive load conditions were counterbalanced through the sessions.

Polysomnography

Polysomnography (PSG) on the habituation and the experimental SF and RS nights was conducted according to standard guidelines defined by the American Academy of Sleep Medicine [56]). Brain (electroencephalogram, EEG), cardiac (electrocardiogram, ECG), ocular (electrooculogram, EOG) and muscular (electromyogram, EMG) activity was recorded at a 256-Hz sampling rate using a Morpheus Polysomnograph (Micromed, Mogliano Veneto, Italy) digital recorder operated with BrainRT software (OSG, Rumst, Belgium). PSG recordings included five channels (scalp locations Fz, C4, C3, Cz and Oz with references A1 and A2), two electrooculograms, anterior and bilateral anterior tibial electromyograms, and two chin EMG electrodes. The participants’ skin was prepared according to standard procedures. Oral and nasal airflow was recorded with a nasal cannula (Reference: 15805-2-NX, Sleep Sense–S.L.P Inc., Elgin, IL, USA) and an oro-nasal thermocouple (Reference: 1472, Sleep Sense–S.L.P Inc., Elgin, IL, USA). Respiratory effort was measured with thoracic and abdominal belts (XactTrace, Natus Medical Inc., Bothell, WA, USA). Snoring was assessed using a Piezo Snore Sensor (Reference: 1250, Sleep Sense–S.L.P Inc., Elgin, IL, USA). Capillary oxygen saturation was monitored with finger-oxymetry (Nonin 8000J Adult Flex Sensor, Medical Inc., Minneapolis, MN, USA). All PSG recordings were exported as EDF+ files and analysed on 21” screens with 30 s polysomnography epochs using PRANA software (PhiTools, Strasbourg, France), both by the main investigator (OB) and a qualified sleep researcher (BD) unaware of the aims of the study.

Sleep Fragmentation procedure

Sleep was fragmented using auditory stimulations at a frequency aimed at mimicking the sleep fragmentation experienced by OSA patients. Sleep fragmentation occurred during 3 consecutive SF nights, in an attempt to make the experiment ecologically closer to what patients with OSA may experience over successive non-restorative nights. The temporal structure of stimulations was based on the fragmentation pattern of age-matched OSA patients previously admitted to the Brugmann Hospital’s Sleep Unit. We adapted to the sleeping needs of each participant (usual number of hours of sleep). On average, it was 7.73 ± 0.62 h per night. For each SF night, the first sleep cycle was left undisturbed, then sleep fragmentation was induced using arousing auditory tones delivered via loudspeakers. In order to prevent participants from habituating to the sounds or their repetition, auditory tones were generated at random intervals between 60s and 120s, with a random alternation between a beep tone and firecracker sounds. The volume was adapted to each participant’s auditory sensitivity as our aim was not to wake them up but to prevent consolidated/restorative sleep. Auditory stimulation was first presented at a low intensity level, and the volume was then gradually increased until microarousals appeared, defined as abrupt shifts of EEG frequency including alpha, theta and/or frequencies greater than 16 Hz, lasting for at least 3 s [57]. At any awakening sign as defined by the AASM (e.g., the presence of an alpha wave pattern lasting at least 30 s), the generation of the auditory tones was manually interrupted until the participant resumed a deeper stage of sleep (N2, N3 or REM) for at least 2 min before resuming the SF protocol. Stimuli were generated in stable N2, N3 and REM states. Scoring of arousals during REM required a concurrent increase in submental EMG lasting at least 1 s [56]. In both the SF and RS nights, participants were informed that auditory tones may be presented with no intention of waking them up, but they were kept unaware which nights and how many nights this would occur. For each night when in bed, they were presented with an example of both sounds used to ensure the good functioning of the auditory stimulation device. No information about the sleep condition was given the following morning, even if participants specifically asked about it.

Statistical Analyses

The experiment used a cross-over, within-subjects design with repeated measures between the SF and RS conditions. Statistical analyses were performed using JASP 0.14.1. (JASP Team; https://jasp-stats.org (accessed on 10 October 2022)). Control (RS) vs. fragmented (SF) sleep effects were investigated using paired *t*-tests or repeated measures analyses of variance after checking for statistical assumptions; if not met, adjustments were made accordingly. When performing multiple comparisons, *p*-values were adjusted using a Holm–Bonferroni correction balancing type I and II errors, and considered statistically significant at an α of 5%.

## 3. Results

### 3.1. Demographic Data and Pre-Experimental Sleep Stability

Demographic variables and scores measured at enrolment are reported in Table 1.

The analysis of the sleep agenda completed every morning during the week preceding the beginning of the experiment evidenced an overall sleep–wake stable pattern as reported in Supplementary Table S1. Only Sleep quality differed across the seven nights (*F*(6, 60) = 2.97, *p* = 0.01). Post hoc tests conducted on consecutive nights disclosed a trend of lower sleep quality on the first compared to the second night (*t*(11) = −3.12, *p* = 0.06). Other self-reported sleep variables (time in bed, sleep onset latency, wake after sleep onset, total wake time, total sleep time, sleep efficiency, wake quality during the day) did not differ across the seven nights (*ps* > 0.1).

### 3.2. Subjective Sleep Quality in SF and RS Conditions

**Figure 3:**
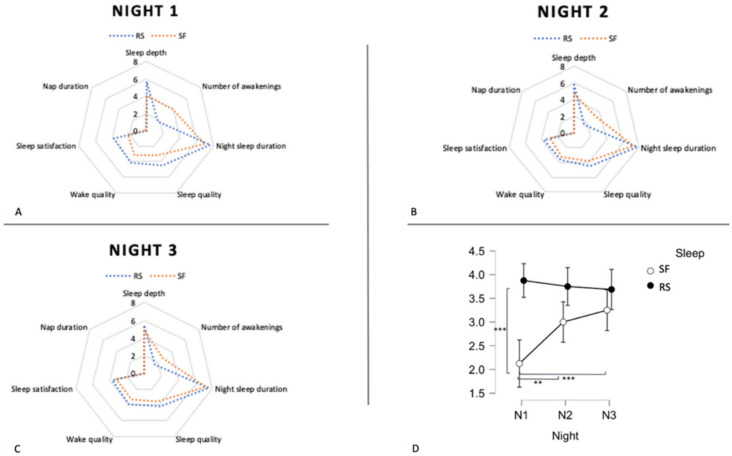
Subjective sleep variables in RS and SF conditions. (**A**–**C**) Sleep parameters for each of the three nights in the RS (blue) and SF (red) conditions. (**D**) Sleep satisfaction across nights and sleep conditions. N1 = Night 1, N2 = Night 2, N3 = Night 3. ** *p* < 0.01, *** *p* < 0.001.

A repeated measures analysis of variance with the within-subject factors Night (N1 vs. N2 vs. N3) and Condition (SF vs. RS) disclosed a Night-by-Condition interaction effect for sleep satisfaction only (*F*(2, 28) = 9.46, *p* < 0.001; all other effects *p* > 0.05). Post hoc comparisons showed participants reporting lower sleep satisfaction after the first night in the SF compared to the RS conditions (*p* < 0.001), and lower sleep satisfaction in the SF condition on the first compared to the second (*p* = 0.01) and the third (*p* < 0.001) nights (Figure 3). No subject napped during the day in both sleep conditions.

### 3.3. Impact of Sleep Fragmentation on Sleep Parameters (PSG)

**Figure 4:**
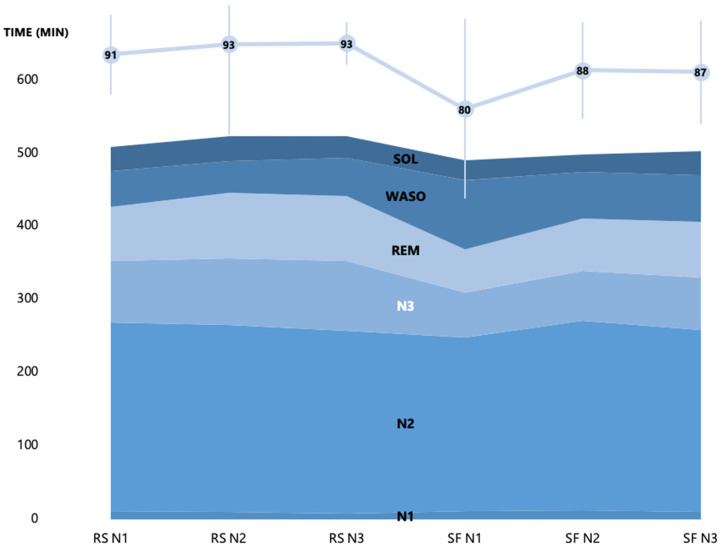
Sleep architecture and sleep efficiency across the three nights in each sleep condition. RS = regular sleep; SF = sleep fragmentation; SE = Sleep Efficiency. SOL = Sleep Onset Latency; WASO = Wake After Sleep Onset; REM = time spent in REM stage; N3 = time spent in N3 stage; N2 = time spent in N2 stage; N1 = time spent in N1 stage.

**Figure 5:**
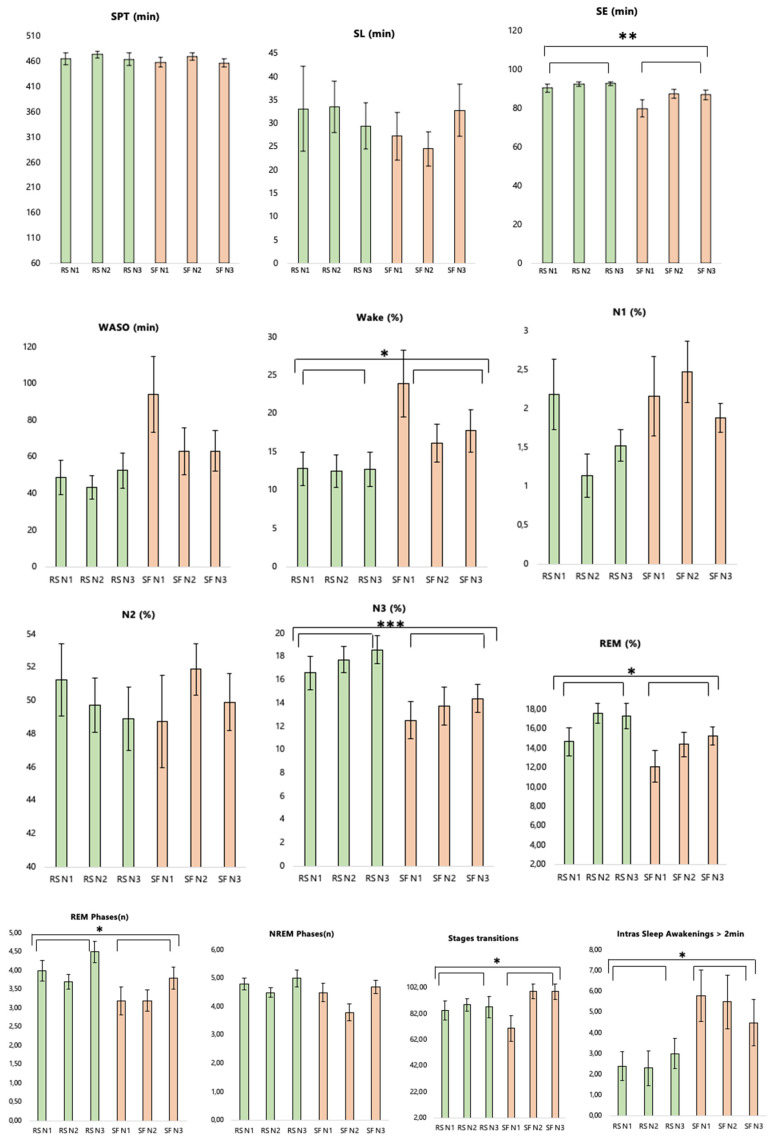
Polysomnographic parameters in control (RS) and fragmented (SF) sleep during the whole night. SPT = Sleep Period Time; SL = Sleep Latency; SE = Sleep Efficiency. WASO = Wake After SleepOnset; Wake (%) = percentage of wake; N1 (%) = percentage of N1; N2 (%) = percentage of N2; N3 (%) = percentage of N3; REM (%) = percentage of REM; REM Phases = number of REM phases; NREM Phases = number of NREM phases; stage transitions = number of stage transitions; Intra-Sleep Awakenings > 2 min = number of wake periods with a minimal duration of 2 min; * *p* < 0.05, ** *p* < 0.01, *** *p* < 0.001. Error bars represent standard errors.

In the SF condition, sleep was fragmented during three consecutive nights (number of delivered stimulations in N1: 98.25 ± 84.34; N2: 163.54 ± 124.48; N3: 212.36 ± 239.18). Sleep duration was similar in the SF (Sleep Period Time [SPT] = 461.88 ± 33.12 min) and RS conditions (SPT = 466.28 ± 41.21 min; *F*(1, 15) = 0.30, *p* = 0.59, *η_p_*^2^ = 0.02), in line with our aim not to shorten sleep duration but induce fragmentation. There was a higher percentage of waking during the night (*F*(1, 13) = 4.81, *p* = 0.05, *η_p_*^2^ = 0.27) and frequency of awakenings longer than 2 min (*F*(1, 9) = 7.39, *p* = 0.02, *η_p_*^2^ = 0.45) in SF than RS, as well as a similar trend for Wake After Sleep Onset (WASO; microarousals/arousals *F*(1, 15) = 3.84, *p* = 0.07, *η_p_*^2^ = 0.20). Sleep fragmentation also altered sleep continuity, as shown by the higher number of NREM (*F*(1, 9) = 5.05, *p* = 0.05, *η_p_*^2^ = 0.36) and REM (*F*(1, 9) = 5.81, *p* = 0.04, *η_p_*^2^ = 0.40) phases in RS compared to SF. The percentage of time spent in N3 (*F*(1, 13) = 27.96, *p* < 0.001, *η_p_*^2^ = 0.68) and REM (*F*(1, 13) = 5.91, *p* = 0.03, *η_p_*^2^ = 0.31) was also higher in RS than SF, while the percentage of time spent in N1 (*F*(1, 13) = 1.52, *p* = 0.24, *η_p_*^2^ = 0.11) and N2 (*F*(1, 13) = 0.009, *p* = 0.92, *η_p_*^2^ < 0.001) was similar between sleep conditions (Figure 4 and Figure 5).

**Figure 6:**
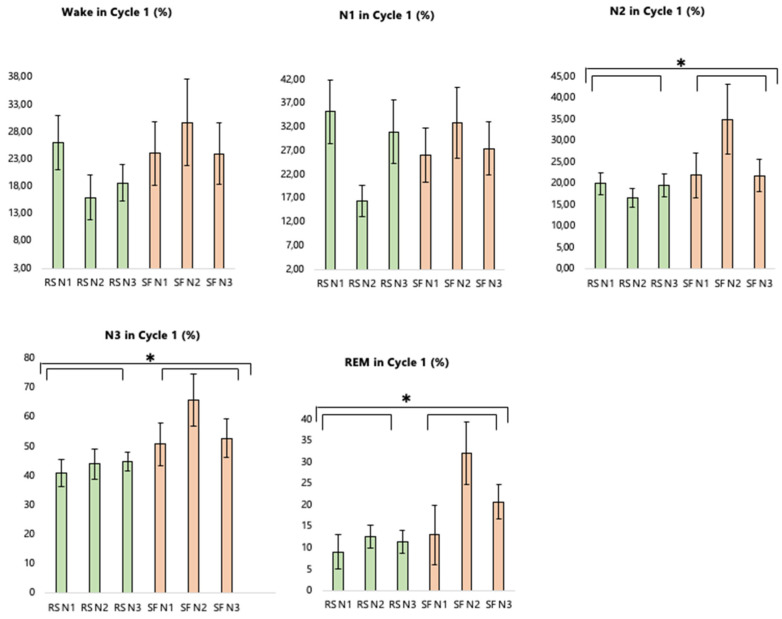
Polysomnographic parameters in control (RS) and fragmented (SF) sleep during the first sleep cycle. Wake in Cycle 1 (%) = percentage of Wake; N1 in Cycle 1 (%) = percentage of N1; N2 in Cycle 1 (%) = percentage of N2; N3 in Cycle 1 (%) = percentage of N3; REM in Cycle 1 (%) = percentage of REM. Error bars represent standard errors. * *p* < 0.05.

In the first sleep cycle (that was kept alike—no stimulation—in both sleep conditions), percentages of N2 (*F*(1, 9) = 5.23, *p* = 0.05, *η_p_*^2^ = 0.37), N3 (*F*(1, 9) = 7.81, *p* = 0.02, *η_p_*^2^ = 0.47) and REM (*F*(1, 9) = 7.31, *p* = 0.02, *η_p_*^2^ = 0.45) were higher in the first cycle in the SF than the RS condition. Sleep latency (SL) did not differ between sleep conditions (*F*(1, 15) = 0.81, *p* = 0.38, *η_p_*^2^ = 0.05). However, sleep efficiency (SE) was lower in the SF than the RS condition (*F*(1, 15) = 8.82, *p* = 0.01, *η_p_*^2^ = 0.37). Percentage of Wake in the first cycle (*F*(1, 9) = 3.47, *p* = 0.10, *η_p_*^2^ = 0.28) and percentage of N1 in the first cycle (*F*(1, 9) = 0.14, *p* = 0.71, *η_p_*^2^ = 0.02) were not different across sleep conditions (Figure 6).

Across the different nights, participants may have started to get used to sleeping in the laboratory, as suggested by an increase in SE over successive nights (*F*(2, 30) = 3.51, *p* = 0.04, *ηp*^2^ = 0.19). Post hoc analyses highlighted a trend of an increase in SE between the first and second/third nights (*ps* = 0.08), and no difference between the second and third nights (*p* = 0.97). This is also in line with the analysis of REM percentage (*F*(2, 26) = 3.68, *p* = 0.04, *η_p_*^2^ = 0.27). Post hoc analysis highlighted a trend of an increase in REM percentage between the first and second/third nights (*ps* < 0.07), and no difference between the second and third nights (*p* = 0.80). Significant changes also appeared between nights for stage transitions (*F*(2, 18) = 5.18, *p* = 0.02, *η_p_*^2^ = 0.16). Post hoc analyses showed increased sleep stage transitions in the first compared to the second/third nights (*ps* = 0.03), and no difference between the second and third nights (*p* = 0.86).

There was also a trend of an interaction between the Nights and Sleep conditions for stage transitions (*F*(2, 18) = 2.99, *p* = 0.08, *η_p_*^2^ = 0.25). Post hoc analyses revealed an increase between the first and second nights of SF (*p* = 0.02) and between the first and third nights of SF (*p* = 0.02), but not between the second and third nights (*p* = 1.00). All other comparisons were non-significant in the control sleep (RS) condition (all *ps* > 0.05).

### 3.4. Neuropsychological Evaluation

**Figure 7:**
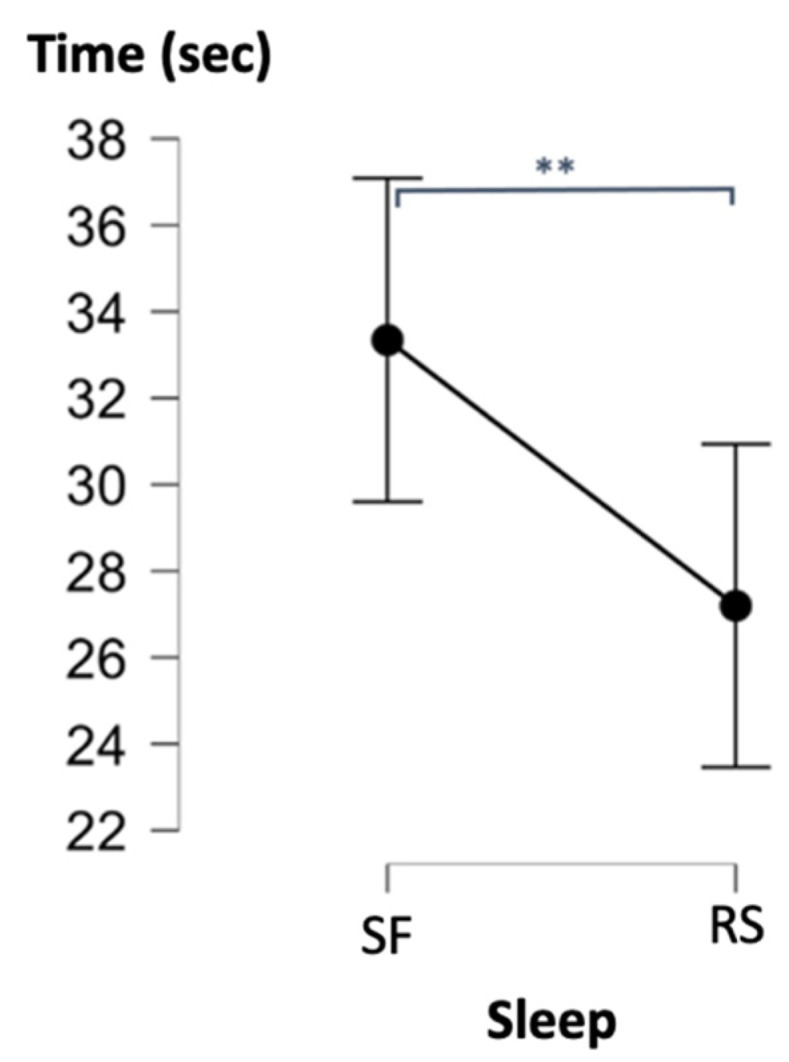
SF-related interference effects (Stroop task). ** *p* < 0.01. Error bars represent standard errors.

Repeated measures analyses of variance of the within-subjects factor Condition (SF vs. RS) and the between-subjects factor Order (first administration in SF vs. RS condition) were conducted on the outcome measures from the neuropsychological tests. Only inhibition (interference effect, i.e., the difference between response speed in the denomination and interference conditions) in the Stroop test was higher in the RS than in the SF condition (*F*(1, 14) = 8.10, *p* = 0.01, *η_p_*^2^ = 0.37; Figure 7). No SF vs. RS condition-related effect was found for all other neuropsychological tests (see Table 2).

### 3.5. Induced Cognitive Fatigue (Subjective Scales and TloadDback Task)

#### 3.5.1. Visual Analog Scales (VAS)

CF was subjectively assessed using visual analog fatigue (VASf) and sleepiness (VASs) severity scales each time before and after the 16 min practice of the TloadDBack task, and visual analog scales for stress and motivation were administered to control for potential confounds.

**Figure 8:**
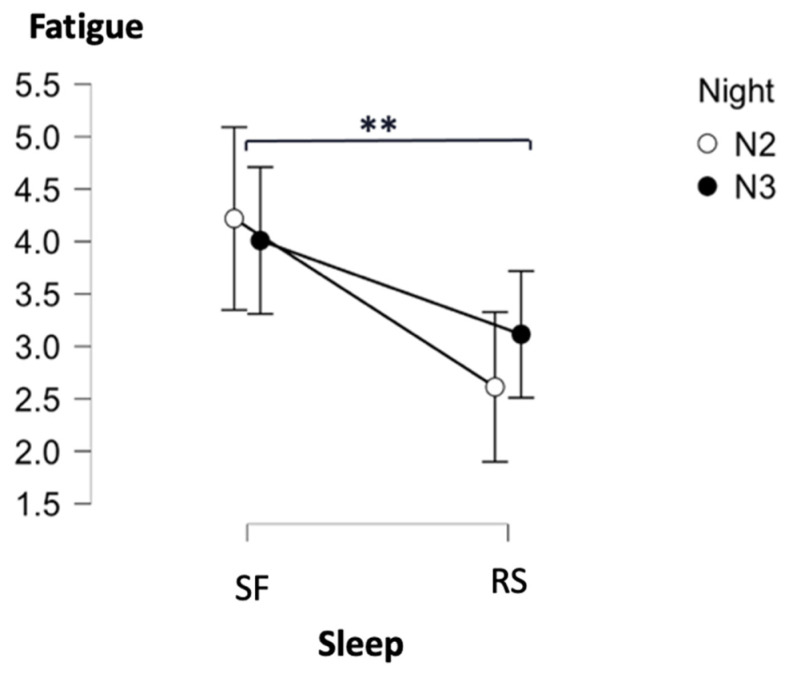
Visual analog scale for fatigue (VASf) prior to the TloadDback task. Note: ** *p* < 0.01. Error bars represent standard errors.

First, we compared VAS scores between the SF and RS conditions prior to the practice on the CF-inducing TloadDBack task. A repeated measures analysis of variance with the within-subject factors Night (N2 vs. N3) and Condition (SF vs. RS) disclosed a main Condition effect with higher subjective fatigue (VASf) in the SF than in the RS condition (*F*(1, 15) = 8.77, *p* = 0.01, *η_p_*^2^ = 0.37; Figure 8). Other effects were non-significant (*ps* > 0.5) (see Supplementary Materials).

To correct for baseline differences and correctly assess the TloadDback-related subjective induction of cognitive fatigue, we computed for each variable a corrected post-task VAS score as a proportion of the pre-task score (bisection of a 10 cm line), multiplied by 100. A repeated measures analysis of variance with the within-subject factors Cognitive Load (LCL vs. HCL) and Condition (SF vs. RS) disclosed no statistically significant effects (*ps* > 0.5).

#### 3.5.2. TloadDback Task

Maximal performance (i.e., high cognitive load ISI) computed after the first experimental night during the calibration session was similar in the RS and SF conditions (RS mean ISI = 775 ± 144 ms [range = 600–1000]; SF mean ISI = 756 ± 136 ms [range = 600–1100]; *p* = 0.25).

**Figure 9:**
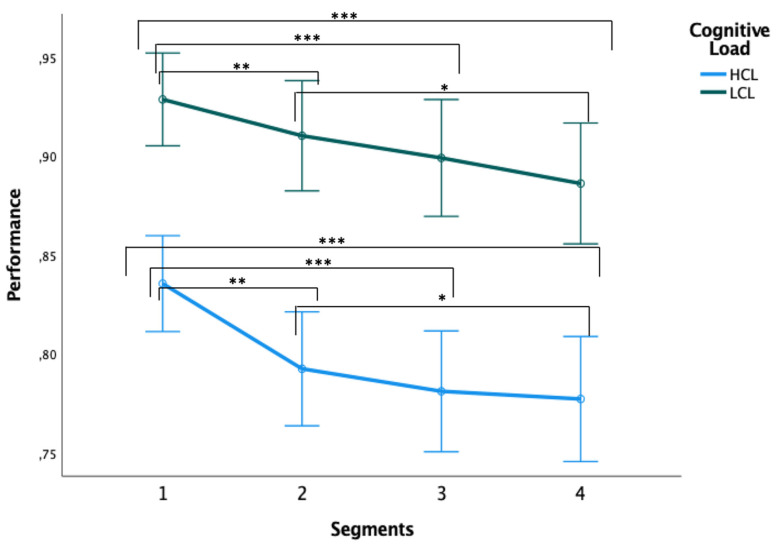
Performance (accuracy percentage) across the four 4 min segments in the 16 min duration TloadDback task. Note: LCL = low cognitive load; HCL = high cognitive load; * *p* < 0.05, ** *p* < 0.01, *** *p* < 0.001. Error bars represent standard errors.

To assess the evolution (and fatigue-related deterioration) of performance during the 16 min practice of the TloadDback Task, accuracy per block was averaged over four successive 4 min long segments, and performance evolution compared between each segment (Figure 9). A repeated measures analysis of variance conducted on accuracy with the within-subject factors Segment (1st vs. 2nd vs. 3rd vs. 4th), Cognitive Load (LCL vs. HCL) and Condition (SF vs. RS) disclosed a main Cognitive Load effect with better accuracy in the LCL compared to the HCL condition (*F*(1, 13) = 34.30, *p* < 0.001, *η_p_*^2^ = 0.73), a main Segment effect with decreased performance starting at the second segment (*F*(3, 39) = 14.41, *p* < 0.001, *η_p_*^2^ = 0.53), and a weak trend towards a main Condition effect with higher accuracy in the RS compared to the SF condition (*F*(1, 13) = 3.42, *p* = 0.09; *η_p_*^2^ = 0.21).

## 4. Discussion

In the present study, participants were exposed both to three consecutive nights of auditory induced sleep fragmentation and three nights of regular sleep, counterbalanced. Our results show the impact of SF on sleep, especially after the first night. Unexpectedly, young healthy participants seemed able to habituate to auditory disturbances on the two following nights, allowing them to partially compensate for the impact of SF-related stimulations. The habituation night performed under complete PSG one week prior to initiating the experiment allowed us to control for a potential first-night effect that could increase the number of awakenings. Indeed, recent studies show that although a first-night effect is present in most cases, it is only in the first night of PSG recording and is less pronounced among young adults [58]. Moreover, counterbalancing the order of sleep conditions with which the experiment began allowed us to further control any potential remaining repercussions of a potential first-night effect, biasing the sleep fragmentation protocol.

Although cognitive load capacity as estimated during the calibration session of the TloadDback task tended to be lower in the SF than in the RS condition, cognitive fatigue (CF), i.e., the ability to sustain accurate performance over practice time, was similarly affected in both conditions. In the present study, we aimed to generate distinct amounts of subjective and objective CF in situations implying uniform task complexity, but whereupon the processing time allowed for ongoing data is controlled to intensify cognitive demands. This control makes particular sense when considering the major inter-individual dissimilarities in net cognitive ability [59], which implies different perceptions of the difficulty of the same task across different individuals. Thus, our method aimed to stabilize learning-related outcomes prior to unbiasedly examining CF effects [28]. Performance during TloadDback practice was divided into four temporal segments to evaluate its evolution through time facing a fatigue-inducing task. Performance gradually diminished across blocks, independently of the sleep condition or the cognitive load. This illustrates the inability to sustain proficient treatment of entering information, and by that the specific and significant effect of the task on triggering CF [28]. This gradual decrease represents a powerful function of the human processing system defined as “the principle of graceful degradation” (i.e., when at least two cognitive processes consume the same limited resources, ensuing decline may occur in performance for the pair or at least one of the active processes) [60]. Therefore, extended cognitive demands would trigger subjective CF and a consequent and steady decline in performance through the 16 min task [28]. Performance across the four blocks was also lower in the high (HCL) than in the low (LCL) cognitive load, supporting the hypothesis that CF secondary to strenuous cognitive demands is guided by the consumption of limited resources [60] when subjects are completing the task at the limit of their aptitude. Thus, subjective CF would intensify prompter than sleepiness, the latter rather characterizing tasks that present lower cognitive solicitations, usually followed by a larger increase of sleepiness than of CF [28]. In the case of the present study, even with the cumulative effect of SF, and when facing a CF task specifically tailored to each subject’s best capabilities, performance evolution was only marginally higher (and non-significant) in the RS than the SF condition. This is in line with previous results, suggesting that SF with unaffected total sleep duration would not markedly behaviourally impact cognitive performance in most domains [61,62,63], whereas neurophysiological measures would be more sensitive to moderate sleep disruption impairments [63].

Usually, SF experiments result in many brief arousals, increasing the time spent in N1 sleep while decreasing the time spent in SWS and REM stages. Previous SF studies thoroughly controlled total sleep time to ascertain that SF-related results are not merely imputable to partial sleep deprivation [64]. Moreover, sleep fragmentation was found to have greater repercussions on sleep quality than sleep restriction [3]. Consequently, our experimental manipulation aimed to alter sleep continuity and efficacy without impacting total sleep time. Our results evidence the validity of our experimental design with a decreased time spent in deeper stages (i.e., SWS and REM) and lower sleep efficiency in the SF than in the RS condition, with a similar total sleep time spent in both conditions. Those changes are congruent with those in patients for which OSA might induce arousals, varying from transient EEG to longer EEG arousals, leading to altered sleep architecture and (micro)awakening periods up to several minutes [65]. In line with patients with OSA, and emphasizing the relevance of continuous restorative, undisturbed sleep, the subjective assessment of fatigue was increased in our participants after SF, although sleep quantity was equivalent between conditions. As mentioned above, our participants seem having started to habituate to SF after the first night, as evidenced by increased SE on the second and third nights. Subjective sleep satisfaction likewise improved over the 3 SF nights. It is worth mentioning that sleep was not fragmented during the first sleep cycle, which may have contributed to allow minimal restorative sleep effects in the SF condition. Still, the first sleep cycle (that was kept alike in both sleep conditions) featured increased N2, SWS and REM stages in the SF condition. This is also in line with increased REM percentage after the first night, as well as stage transitions. Besides sensory habituation to auditory stimulations [65], such adaptation may be explained by a diminished sensitivity developing with accumulating sleepiness as an outcome of sleep fragmentation itself [11,66,67].

Other cognitive aspects (memory, vigilance, inhibition and verbal fluency) were assessed in a neuropsychological battery following the first night in each sleep condition. Although objective PSG and subjective reports evidenced SF-related effects after one night, objective vigilance known to be sensitive to sleep loss (as measured by reciprocal reaction time [53]) was unimpaired, suggesting that young healthy subjects can compensate for the consequences of one SF night. This is in line with previous research showing comparable attentional levels between young patients with OSA and age-matched controls [68]. Particularly as those participants were university students or had higher diploma studies, higher cognitive reserve might have helped them overcome the effects of sleep fragmentation on cognition [69]. Additionally, previous studies found PVT not to be sufficiently sensitive to the effects of moderate sleep disturbances [3]. Accordingly, in another study, we found no OSA treatment-related changes in PVT performance, whereas verbal memory improved after the first night under treatment and remained stable up to three months later [70]. In the executive function domain, however, inhibition performance deteriorated after one night of SF, consistent with previous findings linking sleep deprivation to top-down alterations [71]. When the experiment started with SF, incongruence task and interference (incongruence time–denomination time) durations were significantly longer after the first night of SF than following one night of RS. Altogether, this may indicate a learning effect for this task in both sleep conditions. However, results improving when the experiment started with SF could be understood as an early effect of sleep fragmentation on inhibition. The second assessment following sleep restauration would therefore amplify the increase in inhibition abilities and strategic execution. This is in line with the fact that when the experiment started with restored sleep, inhibition performance did not decrease as expected during the second assessment in SF condition.

This is also consistent with previous research stating that changes in sleep stage progression and important interruption of the normal sleep process would considerably contribute to cognitive deficits [67]. Verbal fluency, both for phonological and semantic tasks, diminished at the second testing time point (e.g., more words were generated in the RS than SF condition, when the experiment started in the RS condition. Similarly, more words were generated in the SF condition than RS condition, when the experiment started in the SF condition). This could be explained by motivation, participants having understood that this was the last task of the one-hour neuropsychological battery and anticipating the upcoming tiring and long task (calibration of the TloadDback task, duration ≈ 35 min). This is coherent with Ackerman’s theory stating that depletion of performance may be explained by a loss of interest in a fatiguing task characterized by time pressure and verbal content, especially when time on task proceeds without breaks [72].

Visual analog scales assessed subjective states (sleepiness, fatigue, stress and motivation) after each step of the experiment. Even though studies found a robust link between sleepiness and fatigue [73,74,75], the results here displayed significant differences across sleep conditions only for the corrected fatigue visual analog scale following the TloadDback. Subjectively, CF increased more following restored than fragmented nights. These results are to be understood relative to the baseline that was not the same in the SF and RS sleep conditions. Indeed, fatigue was already higher following sleep fragmentation and even though it significantly increased following the TloadDback task with (higher subjective assessment of the fatigue in the SF condition), the increase was larger in the RS condition. A possible explanation is that participants were already feeling sufficient fatigue following the SF condition that the task impacted less on the evaluation of their evolution of fatigue. This could be linked to previous studies demonstrating reduced performances in face–motion processing tasks following sleep deprivation and fragmentation [76,77]. In particular, REM sleep fragmentation plays a major role in the processing of emotional information [78], degraded emotional processing [79] and defective affect regulation [80,81]. Taken together, these results illustrate a potential difficulty in analysing overwhelming symptoms following SF that impacted SWS and REM stages. This difficulty when dealing with an emotional processing task was also found in patients with OSA and insomnia [81].

## 5. Conclusions

To sum up, we found that sleep fragmentation altered sleep continuity, even with preserved total sleep time, and disrupted sleep architecture, subjective evaluation of fatigue and performance. However, young subjects proved able to compensate for consequences of altered sleep continuity in several cognitive domains. Only inhibition was affected after a night of fragmented sleep. Even with the cumulative effect of two to three nights of SF, and when facing a CF task specifically tailored to each subject’s best capabilities, performance evolution only tended towards a deterioration in the SF condition. Further studies should investigate the impact of sleep fragmentation in more extended (i.e., up to weeks) and/or pathological (i.e., OSA, Restless Legs Syndrome…) disruption settings.

## Figures and Tables

**Table 1:** Demographic data.

	Data (Mean ± SD)	Range
Age (years)	28.5 ± 4.48	24–38
PSQI global score	3.19 ± 1.33	1–6
BFSm (mental)	3 ± 2.34	0–6
BFSp (physical)	1.94 ± 1.69	0–5
FSS	2.05 ± 0.83	1–3.67
ChQ—Morningness–eveningness	19.5 ± 7.71	8–32
ChQ—Distinctness	16 ± 4.90	10–23
BDI	1.63 ± 1.86	0–6
BAI	4.75 ± 8.33	0–35

Note: Data are mean (±SD) scores. PSQI = Pittsburgh Sleep Quality Index [45]. BFS = Brugmann Fatigue Scale [27]. FSS = Fatigue Severity Scale [46]. ChQ = Chronotype Questionnaire [47]. BDI = Beck Depression Inventory [48]. BAI = Beck Anxiety Inventory [49].

**Table 2:** Outcomes of attentional and memory functions after one night in experimental RS and SF.

	Control Sleep	Fragmented Sleep	Statistics	
PVT-Median	307.19 (27.75)	310.94 (39.17)	t(15) = 0.30	*p* = 0.59
PVT–lapses > 500 ms	0.81 (1.28)	1.69 (3.18)	t(15) = 2.30	*p* = 0.15
PVT-lapses > 2std	2.13 (1.03)	2.25 (1.48)	t(15) = 0.14	*p* = 0.72
PVT-RRT	3.26 (0.29)	3.23 (0.36)	t(15) = 0.26	*p* = 0.62
RLS-15-RM	12.62 (0.88)	12.75 (0.72)	t(15) = 0.48	*p* = 0.64
RLS-15-%RLTC	84.11 (9.15)	87.23 (10.46)	t(15) = 0.81	*p* = 0.43
RLS-15-RD	14.38 (1.09)	14.88 (0.34)	t(15) = 2.07	*p* = 0.10
Digit span (in order)	6.13 (1.20)	6.31 (1.08)	t(15) = 0.90	*p* = 0.38
Digit span (in reverse)	5.31 (1.20)	4.94 (1.24)	t(15) = −1.70	*p* = 0.11
Block tapping	6.31 (1.14)	6.50 (1.21)	t(15) = 0.59	*p* = 0.57

Note: Data are mean (±SD) scores. PVT = Psychomotor Vigilance Task (Basner et al., 2011); RRT = Reciprocal Reaction Time; RLS-15 = episodic memory task; RM = mean of the immediate recall trials, % RLTC = percentage of words recalled in at least two consecutive immediate recall trials from the total number of words recalled, RD = delayed recall; Digit span (in order) = subtest of the WAIS [51]. Digit span (in reverse) = subtest of the WAIS [51].

## Data Availability

Data supporting reported results and Supplementary Materials are publicly available as an OSF project (https://osf.io/ra3wd/).

## Supplementary Materials

The following supporting information can be downloaded at https://www.mdpi.com/article/10.3390/ijerph192315485/s1, Figure S1. Template of the sleep agenda completed during the 7 nights before the first experimental night. Table S1. Subjective reports for sleep the 7 nights before the first experimental night. Table S2. PSG parameters in experimental RS and SF nights. Table S3. Visual analog scales before and after the TloadDback task. Figure S2. Number of generated words for phonological (Figure 2A) and semantic (Figure 2B) subtasks (Verbal Fluency task).
