# Supplementary material for: Impact of Sleep Fragmentation on Cognition and Fatigue
Source: Int J Environ Res Public Health. 2022 Nov 22;19(23):15485. doi: 10.3390/ijerph192315485 (PMC9740245; doi:10.3390/ijerph192315485)
Supplement: Supplementary file 1 [file ijerph-19-15485-s001.zip › ijerph-1993500-supplementary.pdf]

SUPPLEMENTARY MATERIAL

Figure S1. Template of the sleep agenda completed during the 7 nights before the first experimental night.

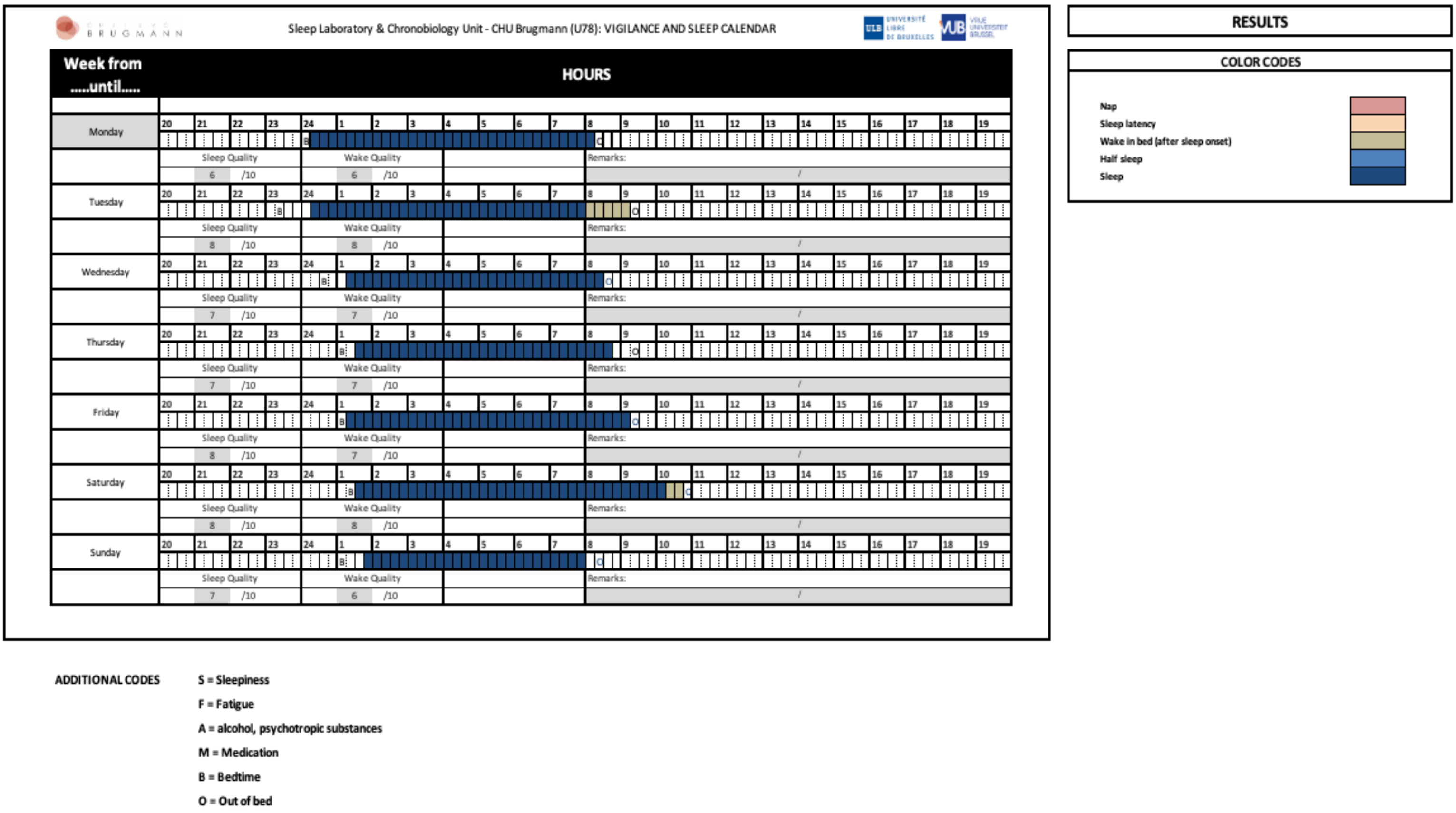

Table S1. Subjective reports for sleep the 7 nights before the first experimental night

	Night 1	Night 2	Night 3	Night 4	Night 5	Night 6	Night 7	Statistics	
TIB	525.00 (54.65)	527.50 (70.31)	548.75 (46.33)	553.75 (54.82)	533.75 (61.50)	513.75 (52.79)	517.500 (71.367)	F(6.66) = 1.05	p = 0.40
SOL	31.25 (35.30)	11.25 (11.30)	16.25 (13.50)	21.25 (24.32)	30.00 (31.33)	17.50 (21.05)	40.00 (41.62)	F(6.66) = 1.67	p = 0.14
WASO	46.25 (71.93)	26.25 (33.31)	36.25 (59.130)	30.00 (27.88)	13.75 (22.58)	22.50 (21.70)	31.25 (38.09)	F(6.66) = 0.90	p = 0.50
TWT	77.50 (102.90)	37.50 (32.93)	52.50 (55.21)	51.25 (40.18)	43.75 (44.07)	40.00 (28.84)	71.25 (77.32)	F(6.66) = 0.92	p = 0.49
TST	447.50 (96.97)	490.00 (65.33)	496.25 (76.61)	502.50 (45.45)	490.00 (66.26)	473.75 (57.73)	446.25 (97.66)	F(6.66) = 1.46	p = 0.21

SE	78.49 (27.99)	86.46 (25.29)	83.23 (25.54)	83.65 (24.32)	84.51 (25.18)	85.57 (25.02)	79.74 (27.30)	F(6.66) = 1.06	p = 0.40
Sleep Quality	6.73 (1.42)	8.09 (1.51)	8.00 (1.61)	7.82 (1.47)	8.09 (1.58)	7.64 (1.50)	7.09 (1.97)	F(6.66) = 3.10	p = 0.01
Wake Quality	7.73 (1.49)	7.55 (1.21)	7.55 (1.44)	7.18 (1.54)	7.55 (1.13)	7.18 (1.33)	6.55 (1.64)	F(6.66) = 2.15	p = 0.06

Note: TIB = Time in bed. SOL = Sleep onset latency. WASO = Wake after sleep onset. TWT = Total wake time. TST = Total sleep time. SE = Sleep efficiency.

Table S2. PSG parameters in experimental RS and SF nights

Polysomnographic parameters	Night	Control Sleep	Fragmented sleep	Directionality
SPT (min)	1	464.45 (44.72)	458.69 (38.86)	RS = SF
	2	469.70 (31.06)	470.16 (29.94)	
	3	464.69 (47.87)	456.78 (32.55)	N1 = N2 = N3
SL (min)	1	33.11 (34.41)	27.22 (20.73)	RS = SF
	2	33.55 (20.95)	24.56 (14.72)	
	3	29.44 (19.79)	32.78 (22.41)	N1 = N2 = N3
SE (%)	1	90.55 (7.79)	79.97 (17.60)	RS > SF
	2	92.60 (4.68)	87.50 (9.44)	
	3	92.80 (4.14)	87.15 (10.11)	N1 = N2 = N3
WASO	1	49.08 (35.24)	94.16 (83.24)	RS < SF
	2	43.48 (34.89)	63.21 (47.99)	
	3	52.63 (38.22)	63.38 (43.70)	N1 = N2 = N3
Wake (%)	1	12.85 (7.70)	23.52 (17.53)	RS < SF
	2	12.51 (8.03)	16.28 (10.56)	
	3	12.76 (7.33)	17.76 (11.19)	N1 = N2 = N3
N1 (%)	1	2.19 (1.80)	2.17 (2.17)	RS = SF
	2	1.84 (1.14)	2.48 (1.70)	
	3	1.53 (0.86)	1.89 (0.78)	N1 = N2 = N3
N2 (%)	1	51.26 (8.59)	48.74 (11.62)	RS = SF
	2	49.76 (6.36)	51.90 (6.66)	
	3	48.91 (7.87)	49.92 (7.20)	N1 = N2 = N3
N3 (%)	1	16.62 (5.72)	12.55 (6.39)	RS > SF
	2	17.76 (4.66)	13.73 (6.93)	
	3	18.61 (4.11)	14.41 (5.03)	N1 = N2 = N3
REM (%)	1	14.68 (5.81)	12.13 (6.55)	RS > SF
	2	17.61 (3.89)	14.41 (5.37)	N1 < N2
	3	17.34 (4.83)	15.27 (3.35)	N1 < N3
Wake in Cycle 1 (%)	1	26.05 (20.77)	24.07 (16.96)	

	2	15.97 (18.66)	29.75 (34.46)	RS = SF
	3	18.68 (10.87)	24.02 (25.25)	N1 = N2 = N3
N1 in Cycle 1 (%)	1	35.31 (30.25)	26.17 (18.15)	RS = SF
	2	16.40 (13.56)	32.98 (32.92)	
	3	31.03 (30.33)	27.52 (23.94)	N1 = N2 = N3
N2 in Cycle 1 (%)	1	19.96 (10.26)	21.97 (17.22)	RS < SF
	2	16.61 (9.36)	35.09 (12.67)	
	3	19.59 (12.67)	21.90 (15.82)	N1 = N2 = N3
N3 in Cycle 1 (%)	1	40.98 (17.50)	50.77 (26.44)	RS < SF
	2	44.03 (20.27)	65.77 (27.39)	
	3	44.91 (14.69)	52.87 (23.37)	N1 = N2 = N3
REM in Cycle 1 (%)	1	9.13 (13.31)	13.05 (16.49)	RS < SF
	2	12.66 (10.92)	32.05 (30.31)	
	3	11.43 (11.56)	20.78 (17.26)	N1 = N2 = N3
Intras Sleep Awakenings > 2min	1	2.40 (2.12)	5.80 (2.74)	RS < SF
	2	2.30 (3.62)	5.50 (5.21)	
	3	3.00 (3.30)	4.50 (4.86)	N1 = N2 = N3
NREM Phases (n)	1	4.80 (0.92)	4.50 (1.27)	RS > SF
	2	4.50 (0.53)	3.80 (1.23)	
	3	5.00 (1.25)	4.7 (0.95)	N1 = N2 = N3
REM Phases (n)	1	4.00 (0.94)	3.20 (1.48)	RS > SF
	2	3.70 (0.48)	3.20 (1.14)	
	3	4.50 (1.08)	3.80 (1.03)	N1 = N2 = N3
Stages transitions	1	84.80 (24.65)	70.90 (17.14)	N1.SF < N2.SF
	2	89.10 (11.87)	99.50 (19.23)	
	3	87.30 (17.22)	99.20 (16.31)	N1.SF < N3.SF

Note: For SPT, SL, SE, and WASO, n = 16. For Wake (%), N1 (%), N2 (%), N3 (%), and REM (%), n = 14. For Wake in Cycle 1 (%), N1 in Cycle 1 (%), N2 in Cycle 1 (%), N3 in Cycle 1 (%), REM in Cycle 1 (%), Intra Sleep Awakenings, NREM Phases, REM Phases, and Stage transitions, n = 10. Data are given as mean (± SD).

SPT = Sleep Period Time. SL = Sleep Latency. SE = Sleep Efficiency. WASO = Wake After Sleep Onset. Wake (%) = percentage of wake during the scored night. N1 (%) = percentage of N1 during the scored night. N2 (%) = percentage of N2 during the scored night. N3 (%) = percentage of N3 during the scored night. REM (%) = percentage of REM during the scored night. Wake in Cycle 1 (%) = percentage of Wake during the scored first cycle. N1 in Cycle 1 (%) = percentage of N1 during the scored first cycle. N2 in Cycle 1 (%) = percentage of N2 during the scored first cycle. N3 in Cycle 1 (%) = percentage of N3 during the scored first cycle. REM in Cycle 1 (%) = percentage of REM during the scored first cycle. Intra Sleep Awakenings > 2 min = number of wake periods with a minimal duration of 2 minutes. NREM Phases = number of NREM phases. REM Phases = number of REM phases. Stage transitions = number of stage transitions.

For all combinations of sleep conditions and cognitive loads, comparisons between VASf prior to and following the TloadDback task confirm that the task subjectively induces cognitive fatigue.

Table S3. Visual analog scales before and after the TloadDback task

	VASf		VASs		VASst		VASm	
	Pre-Task	Post-task	Pre-Task	Post-task	Pre-Task	Post-task	Pre-Task	Post-task
RS LCL	2.74 (2.10)	3.99 (2.08) **	4.13 (2.34)	4.19 (2.50)	1.33 (1.38)	4.19 (2.50)***	7.20 (2.00)	6.18 (2.64)**
RS HCL	2.99 (2.24)	4.28 (2.60) *	3.71 (2.08)	3.25 (2.32)	1.02 (0.95)	1.45 (1.50)	7.08 (2.03)	6.4 (2.54)
SF LCL	4.02 (2.23)	5.12 (1.85)**	4.57 (2.19)	5.46 (2.96)	1.54 (1.91)	1.77 (2.18)	6.64 (2.15)	5.12 (1.85)
SF HCL	4.21 (2.08)	4.89 (2.62)	4.32 (2.63)	3.90 (2.85)	2.05 (2.32)	2.12 (2.41)	6.44 (2.36)	4.89 (2.62)

Note: VASf: Visual analog scale for fatigue. VASs: Visual analog scale for sleepiness. VASst: Visual analog scale for stress. VASm: Visual analog scale for motivation. Data are given as mean (SD). Data is compared between Pre- and Post-task. * p<.05, ** p<.01,*** p<.001.

Additional analysis regarding the order of sleep conditions:

An index aiming at isolating the cost of inhibition by isolating the "interference" variable was computed as denomination time minus interference time, and displayed a similar pattern ($F(1, 14) = 5.81$; $p = 0.03$; $\eta_p^2 = 0.29$). Post-hoc analysis revealed significant difference of interference time between fragmentation (mean = 33.58 ± 8.57) and restored (mean = 22.23 ± 12.57) nights only when the experiment was started with SF ($p = 0.01$). Results suggest a learning effect for this task, particularly when the experiment started with fragmented sleep. The second assessment therefore happening following sleep restauration would amplify the increase in inhibition abilities. This is in line with the fact that when the experiment started with restored sleep, inhibition performance did not decrease as expected during the second assessment in fragmented sleep condition.

Verbal fluency (divided onto a phonological and a semantic part) is another executive function for which the interaction between order of sleep conditions and sleep played a crucial role. Phonological verbal fluency appeared to be statistically different when taking into account that interaction ($F(1, 14) = 22.38$; $p < 0.001$; $\eta p2 = 0.62$). Post-hocs analysis reveal significant differences between fragmentation (mean = 15.25 ± 5.20) and restored (mean = 25.75 ± 4.65) nights only when the experiment started with restored sleep ($p = 0.004$), and between fragmentation nights when the experiment started by fragmented (mean = 25.00 ± 8.04) or restored sleep (mean = 15.25 ± 5.20) conditions ($p = 0.009$).

Semantic verbal fluency also appeared to be statistically different when taking into account the interaction between sleep condition and order of sleep condition ($F(1, 14) = 39.22$; $p < 0.001$; $\eta_p^2 = 0.74$). Post-hocs analysis revealed significant differences between fragmentation (mean = 23.25 ± 6.52) and restored (mean = 34.63 ± 10.77) nights when the experiment started with restored sleep ($p = 0.004$), and between fragmentation nights when the experiment started by fragmented (mean = 34.63 ± 4.69) or restored (mean = 23.25 ± 6.52) sleep conditions ($p = 0.01$). Significant differences were also displayed between fragmentation (mean = 34.63 ± 4.69) and restored (mean = 22.13 ± 5.52) nights when the experiment started with fragmented sleep ($p = 0.002$), and between restored nights

when the experiment started by fragmented (mean = 22.13 ± 5.52) or control (mean = 34.63 ± 10.77) sleep conditions ($p = 0.009$). Both verbal fluencies displayed the same pattern (i.e., a diminution of the number of generated words during the second session). This could be explained by a drop in the motivation, participants having understood that this is the last task of the one-hour neuropsychological battery and anticipating the upcoming long task (calibration of the TloadDback task, duration ≈ 35 minutes).

Figure S2. Number of generated words for phonological (Figure 2A) and semantic (Figure 2B) subtasks (Verbal Fluency task).

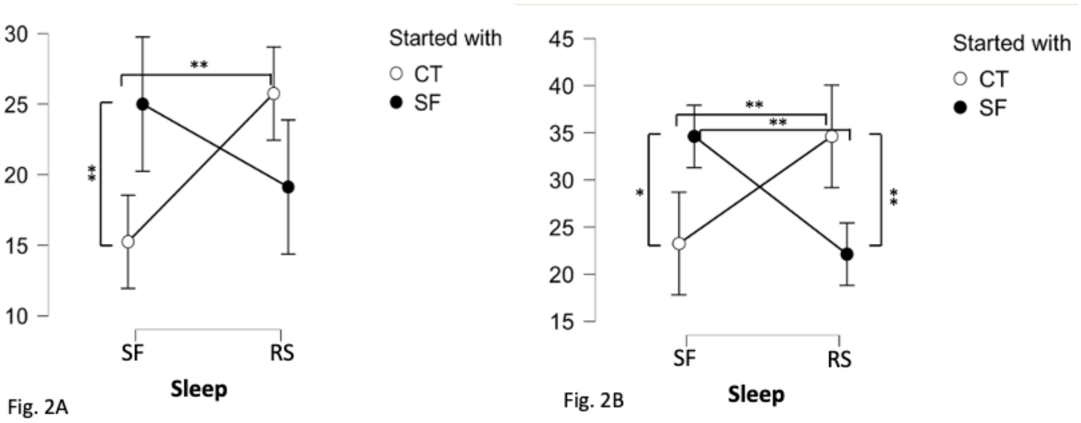

Note: Started with = order with which the experiment began, as sleep condition was counterbalanced within participants. * p<.05, ** p<.01, *** p<.001

After the first night in each sleep condition, a calibration was ran to determine the individual' maximal processing speed (i.e., interstimulus interval, ISI) to later adapt the TloadDback task to its own capacity. The interaction between sleep condition and order of sleep conditions was statistically significant for the ISI ($F(1, 14) = 86.04$; $p < 0.001$; $\eta_p^2 = 0.86$). Post-hocs analysis revealed significant differences between fragmentation (mean = 0.70 ± 0.13) and control (mean = 0.85 ± 0.12) nights when the experiment started with control sleep ($p < 0.001$), and between fragmentation (mean = 0.83 ± 0.15) and control (mean = 0.69 ± 0.08) nights when the experiment started by fragmented sleep ($p < 0.001$). This illustrates an effect linked to task repetition, participants being able to hold an accuracy > 85% with shorter stimuli interval at the second ISI calculation, independently of their sleep condition. This could be interpreted in the framework of offline consolidation largely characterized for motor learning paradigms (Robertson, 2009). Therefore, providing ample task practice (through the four steps of the calibration phase) and a stabilization of learning-associated impacts (the cognitive fatigue inducing task starting the following morning) was an essential prerequisite for investigating CF effects on performance (Borragán, 2016). Moreover, the task repetition effect during the TloadDback task was controlled at calibration level as for the following days of experiment, the ISI implemented during the 16min-task was always associated to the one emerging from the last calibration in the same sleep condition (i.e. a different ISI in each sleep condition).
